# Development of a scalable weight loss intervention for low-income workers through adaptation of interactive obesity treatment approach (iOTA)

**DOI:** 10.1186/s12889-018-6176-0

**Published:** 2018-11-16

**Authors:** Rachel G. Tabak, Jaime R. Strickland, Richard I. Stein, Hank Dart, Graham A. Colditz, Bridget Kirk, Ann Marie Dale, Bradley A. Evanoff

**Affiliations:** 1 Prevention Research Center in St. Louis, The Brown School, Washington University in St. Louis, 1 Brookings Dr, St. Louis, MO 63130 USA; 2 Division of General Medical Sciences, Department of Medicine, Washington University School of Medicine, 4523 Clayton Avenue, Campus Box 8005, St. Louis, MO 63110 USA; 3 Center for Human Nutrition, Department of Medicine, Washington University School of Medicine, 4523 Clayton Avenue, Campus Box 8083, St. Louis, MO 63110 USA; 4 Division of Public Health Sciences and Alvin J. Siteman Cancer Center, Department of Surgery, Washington University School of Medicine, Washington University in St. Louis, 660 South Euclid Avenue, Campus Box 8100, St. Louis, MO 63110 USA

**Keywords:** Worksite intervention, Weight loss, Implementation science, Adaptation

## Abstract

**Background:**

Describing how and why an evidence-based intervention is adapted for a new population and setting using a formal evaluation and an adaptation framework can inform others seeking to modify evidence-based weight management interventions for different populations or settings. The Working for You intervention was adapted, to fit a workplace environment, from Be Fit Be Well, an evidence-based intervention that targets weight-control and hypertension in patients at an outpatient clinic. Workplace-based efforts that promote diet and activity behavior change among low-income employees have potential to address the obesity epidemic. This paper aims to explicitly describe how Be Fit Be Well was adapted for this new setting and population.

**Methods:**

To describe and understand the worksite culture, environment, and policies that support or constrain healthy eating and activity in the target population, we used qualitative and quantitative methods including key informant interviews, focus groups, and a worker survey; these data informed intervention adaptation. We organized the adaptations made to Be Fit Be Well using an adaptation framework from implementation science.

**Results:**

The adapted intervention, Working for You, maintains the theoretical premise and evidence-base underpinning Be Fit Be Well. However, it was modified in terms of the means of delivery (i.e., rather than using interactive voice response, Working for You employs automated SMS text messaging), defined as a modification to context by the adaptation framework. The adaptation framework also includes modifications to content; in this case the behavioral goals were modified for the target population based on updated science related to weight loss and to target a workplace population (e.g., a goal to avoiding free food at work).

**Conclusions:**

If effective, this scalable and relatively inexpensive intervention can be translated to other work settings to reduce obesity and diabetes risk among low-SES workers, a group with a higher prevalence of these conditions. Using a formal evaluation and framework to guide and organize how and why an evidence-based intervention is adapted for a new population and setting can push the field of intervention research forward.

**Trial registration:**

ClinicalTrials.gov: NCT02934113; Received: October 12, 2016; Updated: November 7, 2017.

**Electronic supplementary material:**

The online version of this article (10.1186/s12889-018-6176-0) contains supplementary material, which is available to authorized users.

## Background

The current epidemic of obesity in the US and other countries is projected to greatly increase the prevalence of diabetes and other health consequences [1–6]. Obesity is most prevalent among socioeconomically disadvantaged populations, including racial and ethnic minorities [7, 8]. National data show that obesity is strongly associated with low socioeconomic status (SES), which includes those in hourly and working-class jobs and employees with low education [9–14]. SES factors are related to environmental characteristics and risk behaviors that promote obesity, as well as limited access to weight management resources [15–19]. Despite the compelling evidence for the importance of healthy weight, few weight loss interventions have been rigorously tested in low-SES populations [20–22]. Adaptation of existing intervention models is needed to improve reach and sustainability while maintaining the effectiveness shown in interventions such as the Diabetes Prevention Program (DPP) [23].

Worksites are targeted as a priority location for health intervention efforts by employers, the Centers for Disease Control (CDC), and the National Institutes of Health (NIH) [24–29], because they offer an efficient and effective means of delivering and evaluating such programs, and offer opportunities to reach socially disadvantaged groups. Worksites offer ready access to populations, natural structures for social support, and the opportunity to build health promotion activities on existing communication networks. Importantly, there has been minimal evaluation of the effectiveness of worksite-based health promotion among low-SES employees [30, 31], a population with more limited access to and participation in worksite wellness programs [30, 32–41]. This may be attributable to a number of barriers such as stress and limited communication to these groups [24, 35, 38, 42–47], particularly for low-wage hospital employees [48, 49].

One program that successful promoted weight loss in a low-income, racially diverse population, Be Fit Be Well (BFBW), was implemented in individuals with obesity and hypertension who received healthcare through community health centers [50, 51]. This lifestyle modification program targeted multiple levels, included dietary messaging around the Dietary Approaches to Stop Hypertension (DASH) diet, and incorporated electronic as well as interpersonal supports; participants received both types of supports. Depending on the participant’s available computer access and comfort with computer use, the electronic supports were delivered either through a Web-based system or through a telephone-based system using Interactive Voice Recognition; the electronic support component did not include personal contact. The interpersonal supports included community health workers and linkages to local resources. Behavioral targets for BFBW included diet, physical activity, and hypertension medication adherence, and outcomes assessed were weight loss, blood pressure control, and quality of life [52]. Overall, BFBW showed significant benefit for weight loss at 24 months [51].

While evidence-based interventions promoting weight loss and chronic disease prevention, such as BFBW, are readily available, they require adaptation for implementation in more diverse settings such as worksite settings with low-SES employee populations. Describing the process of adaptation is critical to advancing implementation science [53–56]. The application of a formal framework to an intervention and its use in another setting can advance implementation efforts by shedding light on the steps taken and the modifications required. This can help important stakeholders (e.g., other investigators, practitioners) understand why and how an intervention being implemented is different from the original intervention, and how they might further adapt the intervention for other settings and populations. For the current study, the BFBW program was adapted to fit a worksite intervention targeted at low-SES employees. This manuscript aims to use the model developed by Stirman et al. [55–57] to describe in detail the adaptation and process for adapting BFBW to enhance the external validity and transparency of the adapted intervention, Working for You (WFY) [55].

## Methods

**Fig. 1:**
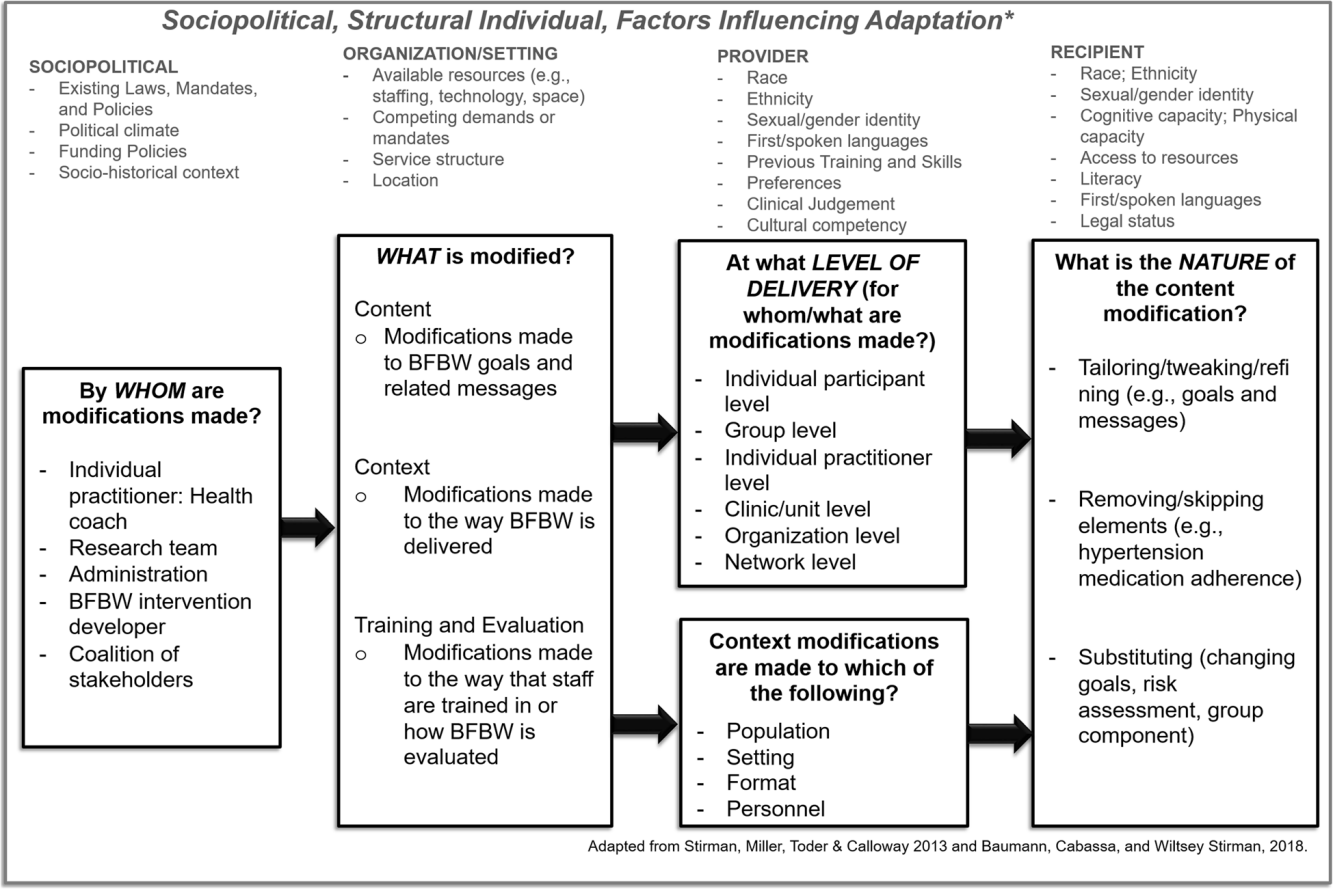
Framework guiding intervention adaptation

To put the adaptations made to BFBW in context, and inform others seeking to modify evidence-based weight management interventions for new contexts and populations, we used the adaptation framework developed by Stirman et al. [55–57] This framework was selected as it is recommended by Chambers et al. [54] as a method to capture adaptations to a specific evidence-based intervention. This framework was developed based on a review of articles describing modifications to evidence-based practices across a variety of interventions and settings [55, 57]. The most relevant components of the framework are summarized in Fig. 1, and these include four Contextual modifications (e.g., change in setting or format), three Content modifications (e.g., adding or omitting components), six levels at which modifications could occur (e.g., consistently throughout a system or organization or only for particular clients), and a code for modifications to Training or evaluation processes. Content modifications can be made at multiple levels.

We first assessed differences in the population and social setting that could affect the success of WFY so that modifications could be made accordingly. This includes changes in motivation for and barriers to engagement and sustainability. Exploratory, iterative, qualitative and quantitative methods including key informant interviews, focus groups, and a worker survey were used to describe and understand the worksite culture, environment, and policies that support or constrain healthy eating and physical activity in the target population (Table 1) [48, 49]. For all of these study components, our target audience was low-wage and hourly workers; therefore, in order to reach these groups, we recruited within specific hospital departments including housekeeping, food service, registration, and patient transport. We distributed flyers and sent emails to recruit focus group participants and worked with department managers to distribute surveys to employees. We interviewed some of those managers as well as members of the corporate wellness committee. All interviews and focus groups were conducted by experienced research team members.

**Table 1:** Summary of formative work to inform intervention adaptation

Method	Participants	Topic	Key Findings
Survey [49]	Housekeepers, food service workers, patient care technicians, registration clerks, medical records clerks, and Emergency Medical Services (EMS) workers *N* = 219	Diet, physical activity, general health, health-related work productivity, commuting, work schedule, work culture and organization, hours worked, dietary history, attitudes toward behavior change, effects of work environment on eating and exercise habits, and current participation in workplace programs	Among this population, 47% had BMI (Body Mass Index) > 30; 64% report trying to lose weight; 27% report 20+ minutes of vigorous exercise more than 2 days per week; 62% sometimes or often drink more than 16 oz of sugary drinks a day.
Key informant interview [48]	Human resource managers and wellness committee members *N* = 5	Barriers and facilitators to implementing wellness initiatives	Communication difficulties are a major barrier to implementing wellness efforts (e.g., many workers were unaware of the wellness initiatives offered by their employer).
Focus groups 1 [48]	Housekeepers, patient care technicians, unit secretaries *N* = 4 groups; 20 individuals	Barriers and facilitators to participation in wellness initiatives, healthy eating, and physical activity	Communication barriers to participation and healthy eating: night-shift workers had less access to health programs or to healthy food choices, work schedules caused meals to be hurried, and food brought for potlucks and employee appreciation did not provide healthy choices.
Focus groups 2	Patient care technicians, administrative assistants, housekeepers, food service workers, patient transport workers N = 4 groups; 13 individuals	Feasibility and acceptability of communication channels; current eating and physical activity habits at work, preliminary message testing	Text messaging feasible and preferred. Most people purchased food from the hospital cafeteria and/or ate free food brought in by others. Most had physically demanding jobs and did not seek out additional activity during break time. The original intended name of the intervention received negative feedback.
Focus groups 3	Food service workers, housekeepers, patient care technicians, registration clerks, schedulers *N* = 3 groups and 2 one-on-one interviews; 24 individuals	Information to refine workplace goals and feedback on methods and logistics of delivering iOTA in a workplace setting; feedback about physical activity self-monitoring	Refinement needed to goals and message wording to enhance relevance and understanding.

First, we identified targets for modifying BFBW using surveys with 219 hospital workers (e.g., housekeepers, food service workers). The full survey methods and results have been published elsewhere [49]. Briefly, the survey found that workers with irregular schedules were less likely to participate in a worksite health promotion program [49]. These findings were incorporated into the content and context modifications described below, such as the inclusion of SMS text messaging and a workgroup-level intervention. Next, key informant interviews with five staff, including managers and wellness committee members, knowledge and expertise from the interdisciplinary team (described below), and a first round of 4 focus groups with 20 individuals (Focus Groups 1) were used to dive deeper into the modification targets, such as time and/or priority barriers to participation in the workplace programs offered [48]. We recruited a similar sample to that described for the survey; the methods and results for this qualitative work have been described elsewhere [48]. Briefly, experienced research team members used a semi-structured script to guide focus group discussions, which were audio recorded and transcribed. The full list of interview topics are published elsewhere [48], but include: work schedule, healthy eating priority, eating at work, current wellness programs. NVivo 10 software (QSR International Pty Ltd., Melbourne, Australia), was used to analyze transcriptions. Using a phenomenological approach, to find the “essence” or common themes across individual experiences [58], the thematic analysis aimed to answer: 1) “what impacts healthy eating and physical activity” and 2) “what can be modified at the workplace?”, and codes were merged and grouped under main themes [48]. We conducted an additional set of four focus groups to gauge interest and willingness to participate in a text-messaging weight loss program and test preliminary versions of the messages (Focus Groups 2), which included 13 individuals. The focus group topics included current usage of text messaging, workplace eating and physical activity behaviors, perceptions of the proposed intervention, and feedback on message content and wording (focus group discussion guide is available in the Additional file 1). Interviews were not recorded in order to protect potentially sensitive information. In addition, recording and transcription were not necessary since the purpose of the focus groups was to get feedback about the intervention components (e.g. message timing, message content, logistics) as well as to gather additional information to be used in creating goal domains specific to the workplace. Participant responses and notes captured during the discussions were reviewed by the research team and used to make decisions about intervention modifications. This supported the decisions for incorporation of SMS messaging as a communication channel to reach the target population.

During goal and message development, we conducted a third round (Focus Groups 3) of three focus groups (*n* = 24) with hospital employees from workgroups similar to those to be targeted for recruitment in the intervention (focus group discussion guide available in the Additional file 1). We used an iterative process for designing goals and messages based on information gathered during focus groups to inform subsequent focus groups. Consequently, recording and transcription were not necessary and summaries of focus group discussions, rather than systematic analysis of transcripts, were presented to the research team. Meetings of the research team were interspersed between the focus groups so that the responses and notes captured during the groups could be discussed and used to modify the intervention. This was aimed to ensure the goals and messages were relevant and understandable to the new target population. Topics for Focus Groups 3 were: perceptions of proposed intervention methods, additional workplace eating and physical activity questions including use of physical activity trackers for self-monitoring, and discussion around the logistics and feasibility of delivering the intervention in a workplace setting.

## Results

Table 1 includes a summary of the key findings from the formative work. Multiple modifiable barriers to improving workplace health promotion were identified from key informant interviews and Focus Groups 1. The barriers were primarily related to lack of engagement included 1) communication barriers (many workers were unaware of the wellness initiatives offered by their employer); 2) barriers to healthy eating and program participation (i.e., night shift workers had less access to health programs or to healthy food choices, work schedules caused meals to be hurried, and food brought for potlucks and employee appreciation did not provide healthy choices); and 3) the opinion of many workers that their work activities provided sufficient exercise, obviating the need for or making them too tired for leisure time physical activity. Content modifications, such as the tailoring of goals and messages and substituting goals and components, were made based on these findings.

WFY was the product of this formative work. This adapted intervention maintains the theoretical premise and evidence base underpinning BFBW, which includes the importance of social-cognitive and socio-ecological factors [59–66], goal setting, and self-monitoring [52, 67–69]. This was informed by the literature developed by the BFBW trial as well as other important trials such as the DPP. Modifications are organized according to the Stirman model in Table 2. Like BFBW, WFY is a multi-level intervention, but capitalizes on the worksite-based nature of the intervention. This novel aspect of the intervention allowed WFY to maintain much of the theoretical basis from BFBW, but remain feasible for the worksite setting and relevant to the target population. This was particularly informed by findings related to barriers to participation identified in the survey and communication barriers identified in Focus Groups 1. Thus, WFY, like BFBW, includes components at two levels, (1) a participatory workgroup-level intervention designed to impact the entire work group, and available to all, and (2) an individual-level intervention (interactive obesity treatment approach; iOTA), targeting only workgroup employees with obesity.

**Table 2:** Description of how BFBW was modified according to Stirman model

Modification	BFBW	WFY
Who made decision to modify?		
Individual practitioner		–
Team		–
Administrator or supervisor		–
Researcher		Surveys, interviews, and focus groups with the target population and supervisors
Intervention Developer		A member of the BFBW development team included on the WFY workgroup
Coalition of Stakeholders		–
What was Modified		
Content modification		Y (described below)
Context modification		Y (described below)
Training and Evaluation	Community health worker training	Health coach training
Context modifications		
Population	Low-income and racially diverse clinic patients with obesity and hypertension	Low-income and racially diverse healthcare workers with obesity
Setting	Community health centers	Worksite
Format	Interactive Voice Recognition or website with community health worker phone calls	SMS with health coach meetings
Personnel	Physician and community health worker	Health coach
Level of delivery for Content modifications		
Individual participant	Participant selects behavioral goals	Participant selects behavioral goals
Group		–
Individual practitioner		–
Clinic/unit	Community health center	–
Organization	Community health center	Work group within an academic hospital system
Network		–
Nature of the Content modification		
1-Tailoring/tweaking/refining		Goals were tailored to the new setting, population, and mode of delivery
2-Integrating the intervention into another framework		–
3-Integrating another treatment into the intervention		–
4-Removing/skipping elements		Hypertension medication adherence component was removed
5-Lengthening/extending (pacing/timing)	24 months	24 months
6-Shortening/condensing (pacing/timing)	24 months	24 months
7-Adjusting the order of intervention components		–
8-Adding elements		–
9-Departing from the intervention (“drift”)		–
10-Loosening structure		–
11-Repeating components		–
12- Substituting	15 goals, 7 removed– based on updated scientific evidence and target population perspectives	11 goals added – based on updated scientific evidence and target population perspectives
	MyHealthRisk	Baseline survey to inform plan
	Group support sessions	Workgroup level intervention

The workgroup-level component of WFY incorporates the important social and physical environmental factors considered in BFBW, but includes a natural network (co-workers) and environment (workplace) where support and environmental changes can occur (Table 2). This is targeted through a participatory approach using the Intervention Design and Analysis Scorecard (IDEAS) tool [70] and a Human Centered Design approach [71–75] to engage members of the work group in designing and implementing interventions to promote healthy eating and physical activity through changes to the work environment. The remainder of the modifications focus on the iOTA, and are organized according to the Stirman model (Fig. 1 and Table 2).

### Who made the decision to modify

The first set of categorizations within the Stirman model concern who contributed to decisions regarding adaptation. The decisions on how to modify the intervention were ultimately made by the interdisciplinary research team. This team included a dietitian, a behaviorist/psychologist with experience in weight loss studies/ interventions, a health communication expert, an internal medicine physician, and occupational health experts with experience working with the target population. A member of the BFBW research team (original intervention developers) was also on the WFY research team. As described above, however, this was informed by, and conducted iteratively with, feedback and input from the target population. In this case, the providers delivering the iOTA intervention (health coaches) were members of the research team, and so were also involved with making these modifications. Finally, an external technology partner was included to develop and program the technological aspects of the iOTA for the modified intervention. This team made modifications to the following components from the Stirman model: Content, Context, and Training, which are mapped onto the framework by Stirman et al. [57], and summarized in Table 2.

### Context modifications

A number of changes were made in the Context of the intervention (i.e., modifications made to the way BFBW is delivered); Context modifications can be made to Population, Setting, Personnel, and/or Format. As described above, WFY was adapted to low-wage employees at healthcare worksites from an intervention meant for patients with hypertension recruited at community health centers. Despite this difference, both populations are racially diverse and low-income.

For Personnel and Format, the WFY intervention includes personal interaction and electronic, automated interaction, as was the case in BFBW; however, there are far fewer formal personal interactions. While BFBW included (1) 18 individual telephone calls with a community health worker; (2) 12 bi-monthly group support sessions; and (3) tailored social and environmental action planning, WFY includes quarterly meetings with a health coach. Based in large part on the findings from Focus Groups 2, WFY provides participants with information about resources available from their employer, replacing the strategies to increase use of community resources, which was part of BFBW. Additionally, in WFY, participants are nested in their workgroup as described above. While this may seem like a modification to the Content, rather than the Context, this decrease in frequency did not lead to deleting elements beyond those described below. This modification was made both for the purposes of scalability within the hospital employee setting as well as to meet the preferences of participants, who have busy, dynamic schedules, making frequent personal interactions challenging, as identified in Focus Groups 1 and 2.

Also a part of Context in the Stirman model, Format is defined as changes made to the Format or channel of delivery. For several reasons including the findings from the formative work and Focus Groups 2, which identified high rates of SMS texting, but lower rates of access to smartphone apps in the context of the current intervention, WFY is delivered by Short Message Services (SMS) text instead of Interactive Voice Recognition or a web interface as was the case for BFBW. Low-SES populations have high rates of texting use, and interactive texting works well for health message delivery and as a tool for self-monitoring and adherence to interventions in low-income populations [76–81]. In contrast to web-based approaches to providing self-monitoring and feedback, SMS text has the advantages of being easily accessible to all workers, low cost, and quickly viewed. As a self-monitoring tool, text messaging offers a “minimal advice, maximal-contact” program [78]. The asynchronous communication offered by texting is appealing to workers who cannot make personal phone calls while at work, and for those with irregular work hours. Additionally, focus groups (Focus Groups 2) with the target population identified SMS messaging as a feasible and preferred communication channel; most participants reported having a cell phone and text messaging capabilities, but only five of the 36 focus group participants had a smart phone, so preferred SMS messaging to a separate smart phone application (e.g., an iPhone or android app) or web-based application.

The texting program also offers more frequent automated contact, as WFY has fewer personal contacts than BFBW. The SMS intervention in WFY is programed to “touch” participants an average of 5 days per week. The SMS system prompts participants to report their weight and their progress on achieving their goals on a “check-in” day each week, and sends immediate, tailored feedback based on their responses. If a participant is reporting progress on their goal, the SMS system suggests a change to the participant’s goal (i.e., increasing behavior frequency or changing to a different target behavior) in between health coaching sessions. Since an aim of BFBW and WFY was to be sustainable and practical, components that would add increased cost were carefully considered, therefore this automatic feedback was provided instead of individualized goal-progress feedback. In addition, the system sends weekly and monthly tips customized to the goals the participant selected.

### Content modifications

The Stirman model includes 12 ways in which an intervention’s Content can be modified; Table 2 outlines in which of these categories we made Content modifications and briefly summarizes the adaptation. While the electronic interactions in WFY occur through SMS, rather than Interactive Voice Recognition or a web interface (as was the case in BFBW), the self-monitoring (Content) was designed with the same key principles in mind: “(1) be easily utilized in a short period of time; (2) include clearly discernible targets; and (3) track easily recallable information” [51, 52]. As with BFBW, the goals were designed to be concrete and to promote an energy deficit and adherence to the intervention protocol [51, 52]. However, these goals were Tailored to the WFY population (Individual participant level) and setting (Organization level), and involved some Substitution. The modifications to the goals (described in Table 2 under Nature of the content modification: substituting) incorporated findings from Focus groups 1 and were refined by the subsequent rounds of focus groups, which presented members of the target population with potential messages, as well as updated scientific evidence, as determined by the interdisciplinary team described above.

Nineteen behavior-based weekly goals were developed for WFY, 8 of which were adapted from BFBW. During the WFY intervention, participants will choose three of these goals during personal health coaching interactions. This is one area in which the Content was modified in terms of Removing core components; in BFBW, participants had hypertension, and were therefore required to select a goal related to hypertension management. In WFY, hypertension was not part of the inclusion criteria or intervention. The interdisciplinary team developed at least 15 tips for each of the 19 goals, which aimed to build self-efficacy, provide motivation, and deliver strategies to support behavior change. Where the team had concerns regarding the fit or comprehension of the tip content or wording, sample language was presented to potential participants for refinement and feedback. To facilitate self-monitoring, participants in both interventions were provided a device for physical activity monitoring (BFBW: pedometer; WFY: Fitbit). The other departure from BFBW was not including “MyHealthRisk”, which is a web-based health risk assessment [82]. Instead, WFY included a baseline survey assessing risk behaviors, which was used to develop a personalized plan; participants receive a report based on their survey responses to review at their first coaching session.

## Discussion

We modified the evidence-based weight and hypertension BFBW intervention to provide a scalable weight loss intervention in the context of a worksite setting with low-wage employees who did not necessarily have hypertension. This process led to an intervention that should be able to be sustainably implemented in workplace settings and also promotes healthy weight behaviors in low-SES worker populations. To allow comparison of study results and to inform future intervention efforts, it is important to formally map the modifications made to this intervention for the new setting and population [54]. While considerable attention has been paid to cultural adaptation, adaptation for implementation has not been well documented [55–57]. Although intervention adaptation is common, it is uncommon to describe the process and theoretical underpinning. Mapping such changes onto an adaptation framework [56] contributes to the growing implementation science literature [53, 54]. This adaptation process fits with other concepts guiding intervention design, including methods such as Intervention Mapping [83], which has been used within worksite health promotion [84–86] to improve intervention fit [87, 88].

Relations of worksite factors with obesity and chronic diseases such as diabetes have been seen elsewhere in the literature [13, 89]. .The employee preferences identified in the current study and incorporated into the intervention adaptation have been identified in other employee populations [90, 91]. Factors identified in the formative work for the current adaptation process such as long work hours and hostile work environments have been found to be associated with obesity [13] and diabetes [89], highlighting the importance of incorporating these as intervention targets in an adapted intervention [91].

This study has limitations worth noting. For this study, we followed Chambers and Norton’s guidance and applied the model by Stirman et al. [54, 57] However, there are several models for adaptation available [88, 92, 93], and the results obtained might have been different were another model selected. Regardless of the model used, we believe formally documenting the steps required for adaptation leads to more reproducible science and improved interventions. While we employed a framework to systematically document how BFBW was adapted to WFY with the aim of enhancing the generalizability of this experience, the study was conducted within only one hospital system in one city in the United States. Therefore, while the approach is likely to be generalizable, the findings from the resulting WFY intervention may not be [54].

The WFY intervention is currently being tested in a large group-randomized trial. As described above, the individual-level intervention is nested within a workgroup-level intervention in which employees participate for 2 years. Employees with obesity in the workgroup are eligible for the individual-level intervention. Survey measures and height and weight are measured at baseline and (all but height) at 6-month, 12-month, and 24-month follow-up. The primary outcome is weight; secondary outcome measures include dietary and physical activity behaviors as well as work satisfaction. In total, 22 workgroups (11 intervention and 11 control) will be enrolled, and approximately 308 participants with obesity (of 990 total) are expected to participate in the individual-level intervention.

## Conclusions

Attention to how and why an evidence-based intervention is adapted for a new population and setting can inform others seeking to modify evidence-based weight management or other health interventions. This has particular relevance if the adapted intervention is found to be effective. WFY is designed to be scalable and relatively inexpensive, such that transparency in the adaptation process may make this intervention easier to translate to other work settings to reduce obesity and diabetes risk among low-SES workers, a group with a higher prevalence of these conditions.

## Additional file

Additional file 1: Data collection tool. Focus group discussion guide. (DOCX 30 kb)

## Abbreviations

BFBW: Be Fit Be Well
BMI: Body Mass Index
CDC: Centers for Disease Control
DASH: Dietary Approaches to Stop Hypertension
DPP: Diabetes Prevention Program
EMS: Emergency Medical Services
HWPP: Healthy Workplace Participatory Program
NIH: National Institutes of Health
SES: Socioeconomic status
SMS: Short Message Service
WFY: Working for You
