# Supplementary material for: Development of a scalable weight loss intervention for low-income workers through adaptation of interactive obesity treatment approach (iOTA)
Source: BMC Public Health. 2018 Nov 16;18:1265. doi: 10.1186/s12889-018-6176-0 (PMC6240310; doi:10.1186/s12889-018-6176-0)
Supplement: Supplementary file 1 — Data collection tool. Focus group discussion guide. (DOCX 30 kb) [file 12889_2018_6176_MOESM1_ESM.docx]

**Additional File**

Focus group 2 script

| Domain | Questions | Follow-ups/probes |
| --- | --- | --- |
| Current text messaging usage | Who has a text-messaging plan?  Who has a smart phone?  Are you allowed to use your phone to text message while working?  Have you ever signed up to receive automated text messages (like from a store, a health program, emergency alerts, etc.)? *[Or ever received any that you didn’t sign up for?]* | Are “plans” unlimited? Are there other types?  ***IF APPLICABLE***: What do long texts look like on non-smart phones?  What are rules about texting/cell phone use at work? What if someone needs to reach you? What about breaks? Do you use phone on breaks?  What kind of program? How did those messages look? Did you recognize the program/know what it was about? Did you read it? Did you respond if it asked for a response? |
| Text-messaging intervention (general questions) | We are developing a weight loss program that uses text messaging to motivate participants and track their progress. The program will consist of [DESCRIBE PROGRAM ELEMENTS]. What are your thoughts about this program?  In the program, we would tell the participants in advance what time of day they’ll be getting the messages. How specific do you think time preferences would need to be (8-hour window, 4-hour window, just time of day like morning/afternoon/evening, etc.)?  If you were trying to lose weight, would you be interested in enrolling in a program like we have described? What factors might influence your decision to participate? | - # of messages per week OK? - If you were in this program, how quickly do you think you’d reply to messages? - Would your response time depend on if you are at work or at home?   Would it be better to specify a time NOT to get messages? |
| Content-specific questions | What do you think of when you hear the term, “refined grain”?  What do you think of when you hear the term “whole milk” mean?  What do you think of when you hear the phrase “brisk activity”?  When you hear the word “restaurants” – what do you think of? Does this include fast food?  ***For non-day shift workers***:   - Meal names/times – is “lunch” still lunch when eating mid-shift at 11pm? Is it dinner or breakfast when night shift eats at 8am? - What does “bedtime” mean for shift workers? | Can you give examples? Are there other ways to say this? (e.g. “white grain”)  Can you give examples? Are there other ways to say this? (e.g. “red milk” or “vitamin D milk”)  Does this include fast food? |
| Sample screenshots | **Give screenshots for sample assessment, feedback, and tip examples. Some specific questions are below:**   1. Assessment messages (tags, spacing/timing, split in 2 messages to remind of goal – is this necessary?) 2. Feedback messages (How do you feel about them? Encouraging/ discouraging?) 3. Tip messages: Is a command at the beginning of a message off-putting? |  |
| Eating at work? | How much time do you have during the workday to eat/drink?  How often do you buy food at work (for a meal or snack)?  Where do you buy food at work (cafeteria, vending machine, other?)  How often is there free food for you at work (parties, people bring in leftovers or baked goods, etc.)? | Do you feel that you have enough time to eat a proper snack/meal?  Do people buy food at work twice or more per day? If so, elaborate. Does it vary by shift?  How do you decide where you will buy food?  Do you usually eat free food – why/why not? |
| Physical Activity at work? | How much do you walk as part of your job? | Other than walking, how much do you move around (and/or have other forms of physical exertion) doing your usual job tasks? |

Focus group 3 script

| Domain | Questions | Follow-ups/probes |
| --- | --- | --- |
| Current text messaging and email usage | Who has a text messaging plan?  Who has a smart phone?  Are you allowed to use your phone to text message while working?  Do you have/use a [WORK] email address? If not, do you have a personal email address? | Are “plans” unlimited? Are there other types?  What are rules about texting/cell phone use at work? What if someone needs to reach you? What about breaks? Do you use phone on breaks?  Where do you primarily check your email (phone, comp, ipad)? Do you have easy access to a computer and printer? |
| Physical Activity at work? | How much do you walk as part of your job? | Other than walking, how much do you move around (and/or have other forms of physical exertion) doing your usual job tasks?  **If in sedentary job**: What opportunities do you have to get physical activity during the work day (i.e. walk on break, use stairs instead of elevators, etc.)? Do you do any of these things? |
| Eating at work? | How much time do you have during the workday to eat/drink?  How often do you **bring** food from home to eat at work?  How often do you **buy** food at work (for a meal or snack)?  Where do you buy food at work (cafeteria, vending machine, food truck, other?)  How much does cost matter in your food purchasing decisions at work?  How often is there free food for you at work (parties, people bring in leftovers or baked goods, etc.)?  If you were trying to eat healthier, how would you change your eating habits at work? | Do you feel that you have enough time to eat a proper snack/meal?  Is there a freezer and refrigerator to store food? Other place to keep your food? Is there a microwave? Where do you eat food that you bring?  Does it vary by shift?  How do you decide where you will buy food?  Do you pay attention to price, do you choose what you’re going to eat based on price? Do employees get cafeteria discounts? If so, do you use it?  Do you usually eat free food – why/why not? Would you be willing to pass up free food in order to lose weight or be healthier?  Is there anything that could change at your workplace to support healthier eating? |
| Text-messaging intervention (general questions) | We are developing a weight loss program that uses text messaging to motivate participants and track their progress. The program will consist of [DESCRIBE PROGRAM ELEMENTS]. What are your thoughts about this program?  If you were trying to lose weight, would you be interested in enrolling in a program like we have described?  One specific question we have is how the messages appear/are identified when they come in to you phone. If you ever get msgs from short codes, they usually have identifier in the message to let you know who is contacting you. When you start the program we will have you program in to your phone the number the messages will be coming from. 1) would that help everybody? 2) would it matter if you had the prefix HealthTXT? | - # of messages per week OK? - How would you feel about weighing in weekly and reporting it to your coach via text message? - When/where are you most likely to weigh yourself? Would you feel comfortable using a scale at work? - Would willing would you be to use a paper or app-based log to track your food and exercise? What would be your preferred method for tracking?   What factors might influence your decision to participate (or not participate)? |
| General text tips | A component of the weight loss coaching is that the participants will receive text messages. Some are asking the participant about their weight loss goals, others are more general and motivational/inspirational.  Here is a list of some of the motivational texts we were thinking about using.  [Handout] | Do these make sense?  Do you think any are not relevant?  Are there any you do not like? Why? |
| Activity tracking | Do you use an activity tracker and if so, what brand?  Do you use your phone to track steps or activity time?  Would you be willing to use tracker device as part of study?  Would getting a free activity tracker as part of study participation influence your decision to participate? | - Is it wrist worn or hip clip? - Do you sync it to your phone/computer? - What do you do with that information? - Why do you have a tracker?   If yes, do you always carry it with you?  Would you prefer a wrist tracker, hip tracker, phone?  Would you be willing to use it daily and sync/share the data with us?  Is there anything that would make it difficult for you to use tracker and share data? Any concerns?  Would you consider it a benefit?  Would you consider it too time-consuming?  Too personal/invasive? |
| Initial session time: | All of the info we need to cover upon joining the program will take approximately 2 hours. We’re trying to decide the best way to do this…   - - Would it be better to talk more on the phone and less at in-person meeting – or vice versa?   - How willing would you be to meet in person before/after work for 60 min? 90min?   - We would try to give some info ahead of time, like the consent form – would email be a good way for us to do this? Do you all use email? If not, how would you prefer we get you the info (snail mail, in person, etc.)? |  |
